# Impact of Classic Adrenal Secretagogues on mRNA Levels of Urotensin II and Its Receptor in Adrenal Gland of Rats

**DOI:** 10.3390/ijms241713412

**Published:** 2023-08-29

**Authors:** Karol Jopek, Marianna Tyczewska, Marta Szyszka, Małgorzata Blatkiewicz, Maria Jopek, Ludwik K. Malendowicz, Marcin Ruciński

**Affiliations:** Department of Histology and Embryology, Poznan University of Medical Sciences, Swiecickiego 6 Street, 60-781 Poznan, Poland; mszyszka@ump.edu.pl (M.S.); mblatkiewicz@ump.edu.pl (M.B.); m.kociecka@gmail.com (M.J.); lkm@ump.edu.pl (L.K.M.); marcinruc@ump.edu.pl (M.R.)

**Keywords:** urotensin 2, adrenal gland, expression

## Abstract

Urotensin 2 (Uts2) is a biologically active peptide involved in the regulation of a variety of physiological and pathophysiological processes. In both the human and rat adrenal gland, the expressions of the Uts2 gene and its receptor (Uts2r) have been described. This paper focuses on the description of the hormonal control of the mRNA levels of urotensin II and its receptor in the adrenal gland of the rat, both in vitro and in vivo. The initial in vitro experiments were carried out on freshly isolated rat adrenocortical cells and their primary culture. The obtained results indicated a stimulating PKA-independent effect of ACTH on the *Uts2* mRNA level in the tested cells, with no changes in the *Uts2r* transcript. Subsequent in vivo experiments showed that ACTH-induced adrenal growth was accompanied by an elevated level of the *Uts2* mRNA, with unchanged expression of *Uts2r*. In the other types of in vivo gland growth studied, enucleation-induced adrenal regeneration and compensatory growth of the gland, the mRNA levels of the studied genes showed no significant differences. The only exception was hemiadrenalectomy, which led to a significant increase in *Uts2* mRNA expression level 24 h after surgery. In 12-week-old rats of both sexes, gonadectomy led to a significant increase in the level of *Uts2* mRNA in the adrenal gland, an effect that was prevented by sex hormones’ replacement. No changes in *Uts2r* transcript levels were observed under these conditions. Thus, this study suggests that the regulation of *Uts2* and *Uts2r* mRNA levels differs significantly in the rat adrenal gland. While *Uts2* transcript levels appear to be mainly dependent on ACTH action, *Uts2r* mRNA levels are not under the control of this hormone.

## 1. Introduction

Adrenal glucocorticoid hormones have a crucial function in regulating various physiological processes, including the stress response and immune system suppression. Additionally, they play a significant role in controlling glucose metabolism, primarily by counteracting the effects of insulin [1,2,3]. In mammals, the regulation of the glucocorticoids levels is mainly under the control of adrenocorticotrophin (ACTH) from the anterior pituitary, which is precisely regulated by hypothalamic–pituitary–adrenal axis (HPA) and is self-regulated by negative feedback from cortisol on both the hypothalamus and pituitary levels. The adrenal glands are also regulated by other classic adrenal secretagogues: angiotensin II (AngII) and K^+^ ions. Disruptions in the regulation have significant metabolic implications. Addison’s disease, caused by chronic adrenal hormonal deficiency, manifests with symptoms like decreased appetite and weight loss. Conversely, excessive levels of glucocorticoids result in Cushing’s syndrome, which is characterized by obesity development and its associated consequences. In addition to the classic factors that regulate adrenal function, such as AngII and ACTH, a widely studied group of peptides affecting adrenocortical activity has been discovered. Among others, the group includes adiponectin, orexin, leptin, and neuromedin U. The physiological functions of these peptides have been investigated in many experimental models of the rat adrenal cortex in vitro (freshly isolated adrenocortical cells, primary cultures of adrenocortical cells) as well as in vivo (peripheral and central administration of investigated peptides) [4,5,6,7]. Previously, Albertin et al. (2006) proposed that the group of factors regulating adrenal function can be extended with urotensin II (Uts2) [8].

Uts2 is a cyclic peptide that is composed of 11 amino acids in primates (H-Glu-Thr-Pro-Asp-c[Cys-Phe-Trp-Lys-Tyr-Cys]-Val-OH). This peptide was discovered at the end of the 1960s as a product of the neurosecretory urophysis system of the Gillichthys mirabilis teleost fish, which is analogous to the mammalian hypothalamic–neurohypophysial axis [9,10]. The homologs of Uts2 are found in various mammals and differ in length and amino acid sequence, but a C-terminal cyclic hexapeptide (Cys-Phe-Trp-Lys-Tyr-Cys) is highly conserved. It suggests that this cyclic hexapeptide is responsible for the biological activity of Uts2 [11]. In rats, Uts2 is composed of 14 amino acids (H-Gln-His-Gly-Thr-Ala-Pro-Glu-c[Cys-Phe-Trp-Lys-Tyr-Cys]-Ile-OH) [12]. Uts2 is formed as a result of cleavage of the C-terminal fragment of preprourotensin II (prepro-Uts2). Human prepro-UTS2 is a product of the *UTS2* gene. Alternative splicing of human *UTS2* leads to the formation of two mRNA and protein splice variants. They are composed of 124 (isoform b, NP_006777) and 139 (isoform a, NP_068835.1) amino acids [13]. Prepro-Uts2 undergoes proteolytic cleavage via a urotensin-converting enzyme that has not yet been identified [13,14]. 

The Uts2 prohormone is quite different in humans and rat and lacks the typical cleavage site [12]. Uts2 binds to the G-protein-coupled receptor named GPR14, which is currently called the urotensin II receptor (Uts2r). Some authors have indicated that the binding of Uts2 to Uts2r is irreversible, and the receptor is functionally silent under normal physiological conditions. The receptor function may be activated via the modulation of its expression but not by elevated levels of Uts2 in the blood [15]. Uts2 and its receptor are widely expressed in the in the endocrine system (inter alia pituitary, pancreas, and adrenal glands [14,16,17,18] and cardiovascular system [19]).

Urotensin II and its receptor are expressed in both the adrenal medulla and cortex of human and rat adrenal glands [20,21]. Both *Uts2* and *Uts2r* mRNAs are detected in freshly isolated and primary culture of rat adrenocortical cells [8]. Furthermore, mRNA expression of *Uts2* and its receptor is observed in certain adrenal cortex neoplasms, such as pheochromocytomas, and neuroblastomas [20,21]. Elevated levels of *UTS2* mRNA have been found in human adrenal tumors compared with non-neoplastic adrenal tissues, implicating *UTS2* in the progression of adrenal tumor development and steroidogenesis [22]. *Uts2* and *Uts2r* mRNAs are also expressed in PC12 rat pheochromocytoma cells [23]. Additionally, *UTS2* mRNA is expressed in SW-13 cells, an adrenocortical cancer cell line, which secretes mature UTS2, indicating its potential role as a factor promoting tumor development [20,24]. Studies have demonstrated that treatment with UTS2 promotes the proliferation of SW-13 cells. In line with this information, urotensin II increases the proliferation rate of human adrenocortical carcinoma cells and pheochromocytoma cells in a concentration-dependent manner [20,21,23].

Uts2 circulates in the blood plasma at a very low concentration [25]. A higher level of plasma Uts2 and its enhanced expression in some organs are observed in primary hypertension, atherosclerosis, arterial restenosis, heart failure, and type II diabetes mellitus [26,27,28]. The mechanism of action of Uts2 is mostly described in the cardiovascular system, where the activation of peptide and its G proteins leads to the production of phospholipase C (PLC) and inositol trisphosphate (IP3), thereby causing the release of Ca^2+^ from the sarcoplasmic reticulum. This mechanism has been well reported for the role of Uts2 in vascular smooth muscle contraction [29]. The smooth muscle contraction effect is observed at a very low Uts2 level. Currently, Uts2 is considered the most potent mammalian vasoconstrictor (even stronger than endothelin) [14]. On the other hand, in normotensive rats, Uts2 causes vasodilation [30,31]. The different effects of Uts2 depend on the vascular bed, dose, receptor density and species from which blood vessel is isolated [32,33,34]. Uts2r also activates ERK1/2 kinase [15,35,36]. The involvement of ERK1/2-dependent signaling pathways is well established in regulating key processes such as adrenocortical cell proliferation [37], apoptosis [38], migration [39], and steroidogenesis [40]. This signaling pathway is regulated by classical adrenal secretagogues (ACTH, AngII) and numerous other biologically active peptides. 

To the best of our knowledge, there are currently no reports in the literature regarding the effects of classic adrenocortical stimulants on the expressions of urotensin II and its receptor in the adrenal gland. Considering the above, this paper focuses on the description of the regulation of *Uts2* and *Uts2r* expression by adrenal secretagogues (ACTH, AngII, K^+^) in rat adrenal glands in different in vivo and in vitro experiments. We hypothesized that ACTH is the sole stimulator among those mentioned above that induces alterations in urotensin II expression at the mRNA level.

## 2. Results

**Figure 1:**
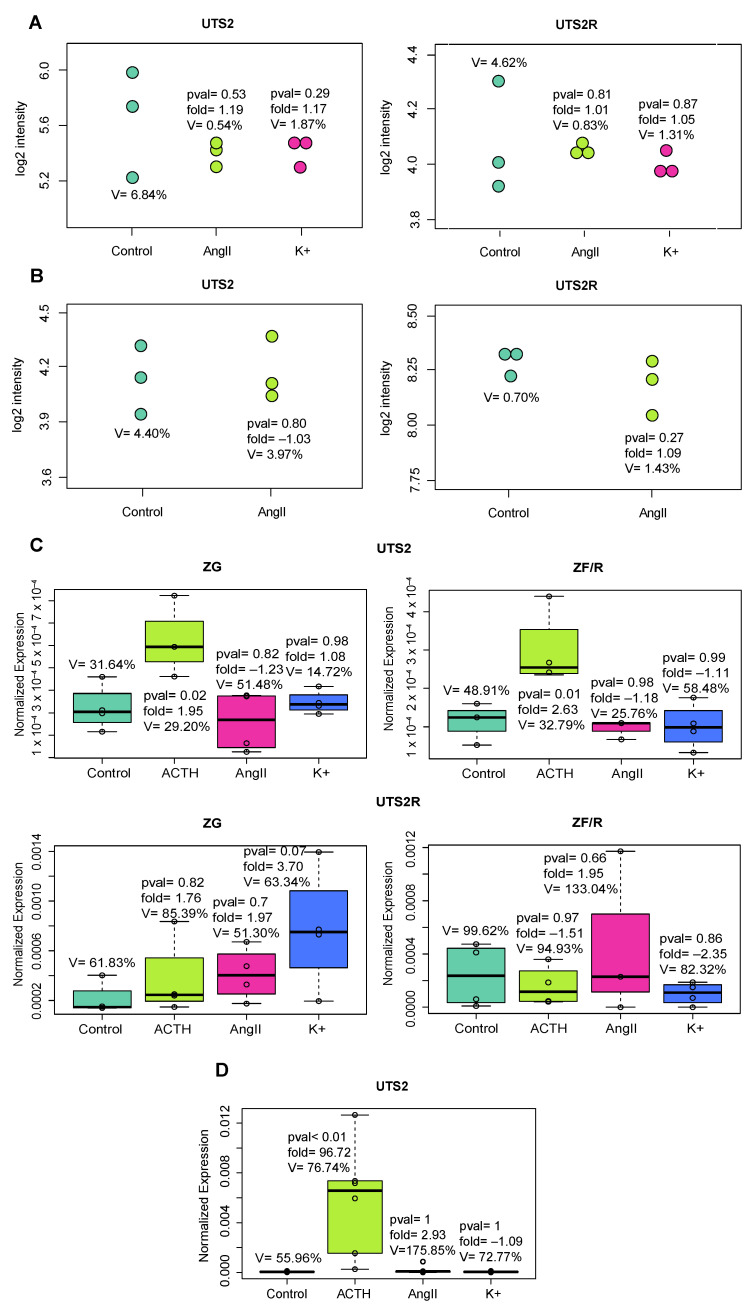
(**A**) The meta-analysis of data deposited by Romero et al. [41] (accession number: GSE8421) that shows lack of any effect of stimulation of freshly isolated rat adrenal ZG cells by AngII (100 nM) or K+ (16 mM) on *Uts2* or *Uts2r* transcript levels. Each dot represents a single log2 signal intensity level extracted from the microarray experiment. The number of biological replicates was three per group. (**B**) The meta-analysis of data reported by Nogueira et al. [42] (accession number: GSE8442). The human adrenocortical tumor cell line (H295-R) stimulated for 1 h with 10 nM AngII did not change *UTS2* or *UTS2R* mRNA levels. Each dot represents a single log2 signal intensity level extracted from the microarray experiment. The number of biological replicates was three per group. (**C**) Bar plot showing the results of qPCR analysis of *Uts2* and *Uts2r* mRNA levels in freshly isolated zona glomerulosa (ZG) and zona fasciculata and zona reticularis (ZF/R) cells stimulated with ACTH, AngII, or K+ (time of exposure 2 h). The number of biological replicates was four per group. The expression level was normalized to *B2m* as a reference gene. The significance levels and folds are shown. (**D**) Potent stimulation of the *Uts2* mRNA level by ACTH (10^−7^ M) in primary culture of rat adrenocortical cells (qPCR analysis). The cells were also stimulated with Ang II (10^−7^ M) and potassium ions (K^+^, 60 mM); however, these secretagogues did not affect the mRNA levels of the studied gene (duration of exposure, 24 h). The expression level was normalized to *B2m* as a reference gene. Significance levels, folds, and coefficients of variation are shown. The number of biological replicates was six per group.

We started our considerations by checking whether the main adrenal secretagogues (ACTH, AngII, and K^+^) can change the expressions of *Uts2* and *Uts2r* transcripts. For this purpose, we used a meta-analysis using publicly available data deposited by Romero et al. in the GEO database (http://www.ncbi.nlm.nih.gov/geo, (accessed on 1 June 2023) accession number: GSE8421) [41]. According to the meta-analysis, subjecting freshly isolated rat adrenal ZG cells to a 2 h exposure of AngII (100 nM) or K^+^ (16 mM) did not show any notable alterations in the levels of *Uts2* or *Uts2r* mRNA (Figure 1). Additionally, another meta-analysis was performed using data reported by Nogueira et al. (accession number: GSE8442; [42]), which indicated that stimulation of human adrenocortical tumor cell line (H295-R) for 1 h with 10 nM AngII did not change the mRNA levels of *UTS2* or *UTS2R* in the studied cells (Figure 1). Both meta-analyses confirmed that AngII and K+ have no effect on *Uts2* or *Uts2r* transcript levels in adrenal glands.

In the next part of the analysis, we therefore focused on the impact of ACTH on the expressions of urotensin II and its receptor. For better readability, we divided our study into two sections: in vitro and in vivo experiments.

### 2.1. In Vitro Experiments

This section describes the direct effect of adrenal secretagogues on the mRNA levels of *Uts2* and its receptor in adrenal glands. We studied the effects of ACTH, AngII, and K+ on the primary culture of rat adrenocortical cells and freshly isolated rat adrenal cells (Figure 1). In the primary culture of rat adrenocortical cells, we observed that ACTH strongly elevated the *Uts2* transcript level. The treatment of cells with K+ as well as AngII did not affect the expressions of the studied mRNAs.

Furthermore, we did not observe any effect of ACTH, AngII, or K+ on the *Uts2r* mRNA level in freshly isolated rat adrenocortical cells. The *Uts2r* transcript level in the cells from the primary culture was below the detection range of qPCR, so we excluded it from the analysis.

**Figure 2:**
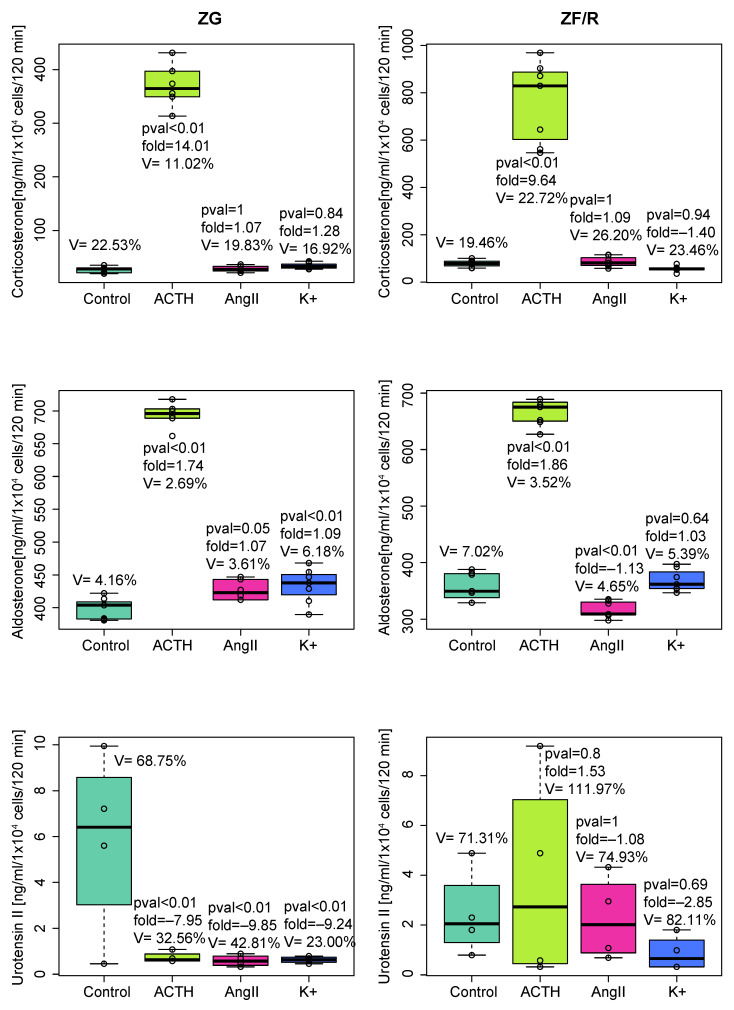
Urotensin II, aldosterone, and corticosterone outputs into incubation medium by freshly isolated ZG or ZF/R rat adrenocortical cells exposed for 120 min to ACTH, AngII, or K^+^. The number of biological replicates was four per group in urotensin II quantification and eight per group in corticosterone and aldosterone determinations. Significance levels, folds, and coefficients of variation are shown.

In the next part of the analysis, we determined the urotensin II concentration in the incubation medium of freshly isolated adrenocortical cells. In the medium, we also quantified the corticosterone and aldosterone levels as a positive control of adrenocortical cell functions. As expected, we observed the ACTH-induced secretion of corticosterone in both ZG and ZF/R and elevated aldosterone secretion by ZG cells stimulated with ACTH, AngII, and K^+^. Surprisingly, the secretion of urotensin II in the ZG cells was downregulated by all secretagogues applied (Figure 2).

**Figure 3:**
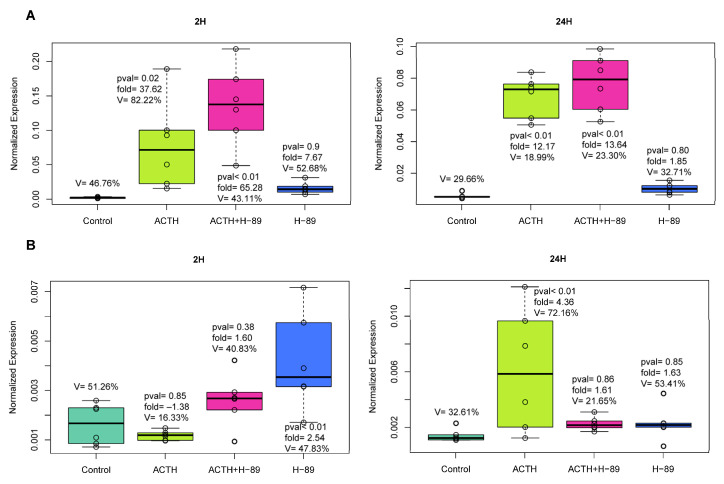
(**A**) The impact of H-89 (10-5M), inhibitor of PKA, on *Uts2* mRNA level in rat primary adrenocortical cell culture. The number of biological replicates was six per group. The significance levels and folds are shown. (**B**) qPCR analysis of the H-89 (10-5M) impact on *Uts2r* mRNA expression level in rat primary adrenocortical cell culture. The number of biological replicates was six per a group. Significance levels, folds, and coefficients of variation are shown.

ACTH acts through stimulation of protein kinase A (PKA). Therefore, to gain insight into the mechanism of *Uts2* mRNA level stimulation via ACTH, in the next part of the analysis, we used H-89, which is an inhibitor of PKA (Figure 3). In this experiment, we observed that H-89 alone slightly but statistically significantly stimulated the level of the *Uts2* transcript. Moreover, H-89 did not inhibit the ACTH-stimulated mRNA level of this gene.

In the same model, we also investigated the *Uts2r* mRNA level (Figure 3). H-89, after a short 2 h stimulation, upregulated the level of the *Uts2r* transcript, but costimulation with ACTH lowered the upregulation range. In the 24 h stimulation period, ACTH increased the *Uts2r* mRNA level, and H-89 diminished the effect.

### 2.2. In Vivo Experiments

In these experiments, we used different in vivo models to follow the changes in the mRNA levels of *Uts2* and *Uts2r* in relation to the duration of exposure to ACTH. Moreover, we checked whether there were differences in the expression of the above-mentioned genes depending on the sex of the rat.

**Figure 4:**
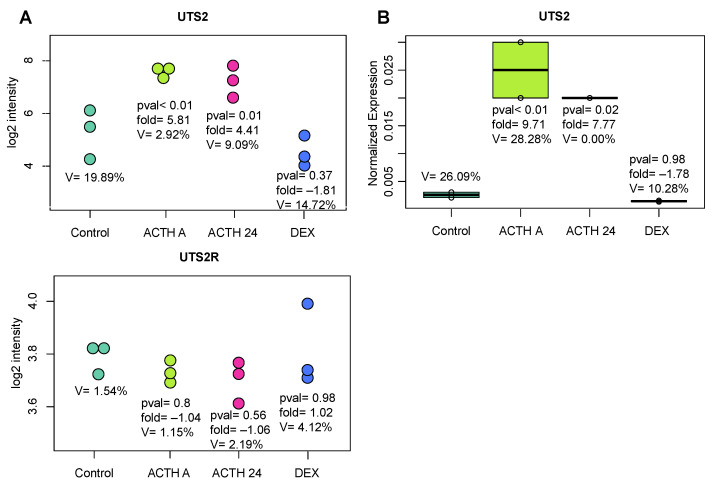
Transcript levels of *Uts2* and *Uts2r* in the rat adrenal gland after ACTH or DEX administration. The rats were administered ACTH for 1 h (acute effect) or 24 h (prolonged effect). The results from microarray analysis (left side of the figure (**A**)) were confirmed by qPCR (right side (**B**)). Significance levels, folds, and coefficients of variation are shown.

The first of the in vivo experiments focused on checking changes in the expressions of *Uts2* and *Uts2r* in the rat adrenal gland after acute and prolonged ACTH administration. The model confirmed that ACTH is the only one of the classic secretagogues that upregulates *Uts2* mRNA level in both acute and prolonged stimulation. Dexamethasone (DEX) administration did not change the *Uts2* or *Uts2r* mRNA levels. Moreover, the level of *Uts2r* transcript in the adrenal gland of studied rats did not change after either stimulation (ACTH) or after DEX administration (Figure 4).

**Figure 5:**
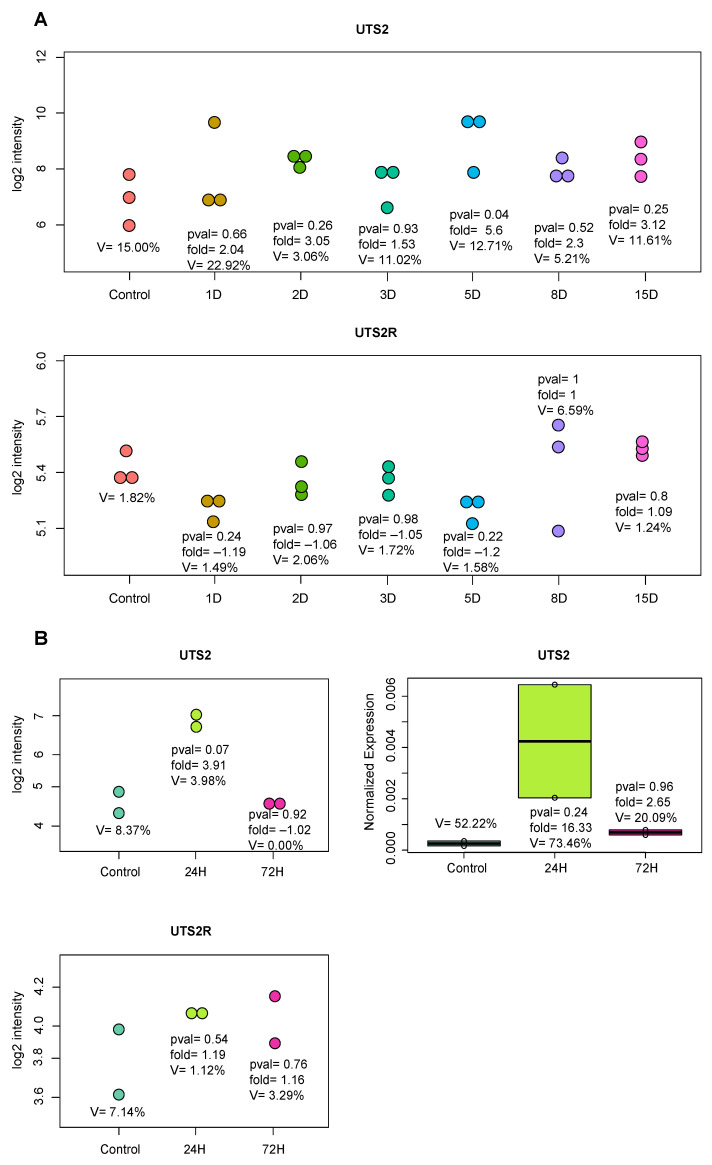
(**A**) Transcript levels of *Uts2* and *Uts2r* in the rat adrenal gland over the course of enucleation-induced regeneration. Each dot represents a single log2 signal intensity level extracted from the microarray experiment. The significance levels and folds are shown. D—day. (**B**) mRNA levels of *Uts2* and *Uts2r* in the rat adrenal gland over the course of hemiadrenalectomy-induced compensatory adrenal growth. Left side of the figure shows log2 signal intensity of gene expression levels from microarray analysis. The number of biological replicates was two per group. The results were confirmed via qPCR analysis, which is shown in the right side of the figure (*n* = 2). The *B2m* gene was used as reference for data normalization. The significance levels and folds are shown.

Another in vivo model that allowed us to check how the expressions of different genes in the adrenal glands behaved over time was enucleation-induced adrenal regeneration. Surprisingly, the *Uts2* mRNA levels tended to be upregulated ay all time points, but the upregulation was statistically nonsignificant. On the other hand, the regeneration downregulated *Uts2r* mRNA levels on days 1 and 5 (Figure 5).

Another experimental model with which we studied the mRNA levels of *Uts2* and *Uts2r* genes was hemiadrenalectomy-induced compensatory adrenal growth. In this experiment, we found that the *Uts2* transcript levels were elevated 24 h after the surgery and returned to control values at 72 h. In the course of the experiment, the *Uts2r* mRNA levels remained unchanged (Figure 5).

**Figure 6:**
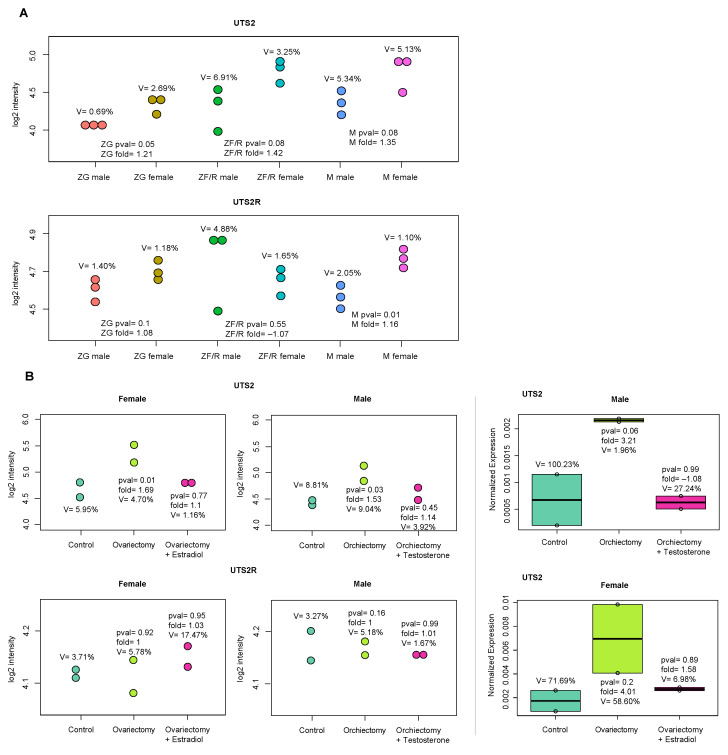
(**A**) Dot plots presenting differences in mRNA levels of *Uts2* and *Uts2r* genes in rat adrenals regarding the sex of the rats and the individual components of adrenals. Each dot represents the log2 signal intensity levels of *Uts2* or *Uts2r*. The significance levels and folds are shown. The number of biological replicates used for the microarray analysis was three per group. ZG—zona glomerulosa, ZF/R—zona fasciculata/reticularis, and M—medulla. (**B**) mRNA levels of *Uts2* and *Uts2r* in gonadectomized rats supplemented with sex hormones. Left side of the figure shows log2 signal intensity of gene expression levels from microarray analysis. *p*-values were calculated in relation to control groups (separately for female and male rats). In each group, *n* = 2. The right side of the figure shows the confirmation of the results obtained from the microarray analysis using a qPCR assay (*n* = 2). The *B2m* gene was used as a reference for data normalization. Significance levels, folds, and coefficients of variation are shown.

In subsequent studies, we examined the expressions of *Uts2* and *Uts2r* in the adrenal glands of male and female rats and attempted to elucidate whether this expression is sex-hormone-dependent. Firstly, we used an Affymetrix^®^ Rat Gene 2.1 ST Array (Affymetrix, Santa Clara, CA, USA) to study the transcriptome profile of the adrenals of adult female and male rats. The results showed that the mRNA level of *Uts2* was slightly lower in male rats than in female rats in all adrenal components (ZG, ZF/R, and medulla (M)). The same applied to *Uts2r* in ZG and M but not in ZF/R (Figure 6).

As subsequent experiments showed, in rats of both sexes, gonadectomy led to an increase in the mRNA level of the *Uts2* gene, and this effect was prevented by the administration of estradiol or testosterone. In the experiment presented here, the mRNA level of the *Uts2r* gene remained unchanged (Figure 6).

## 3. Discussion

As mentioned in the Introduction, Uts2 is a biologically active peptide associated with numerous physiological and pathophysiological processes in the body. Perhaps, its best-known role is in the cardiovascular system, including cardiovascular remodeling, and, particularly, in atherosclerosis, fibrosis, pulmonary hypertension, and congestive heart failure. The role of Uts2 has also been documented in kidney and liver diseases, metabolic syndrome, and type 2 diabetes [26,28]. Uts2 is also involved in the function of the neuroendocrine system, exerting its effects at the level of both the hypothalamus and the peripheral organs, where it exerts either a direct effect on parenchymal cells (mediated by Uts2r) or affects them through the paracrine pathway.

Given the relatively few and inconsistent reports on the role of Uts2 in the regulation of adrenal cortex function, we attempted to clarify the role of the investigated peptide in the function of this endocrine gland in our current study. In the first stage of our study, we conducted a series of in vitro experiments. Previously, Albertin et al. (2006) [8] demonstrated the expressions of *Uts2* and *Uts2r* mRNAs in both freshly dispersed and cultured rat adrenocortical cells and observed the inhibitory effect of Uts2 on basal but not ACTH-stimulated corticosteroid secretion. In contrast, Romero et al. (2007) [41] did not observe effects of AngII or K^+^ on the expressions of *Uts2* and *Uts2r* mRNA levels in freshly isolated rat ZG cells. Additionally, Nogueira et al. (2007) [42] did not identify an effect of AngII on the mRNA levels of these genes in H295-R cells.

In this study, we focused mainly on the impact of classical adrenal cortex secretagogues (ACTH, AngII, and K^+^) on the mRNA levels of *Uts2* and *Uts2r* in the primary culture of rat adrenocortical cells as well as in freshly isolated cells. The obtained results indicated a stimulating PKA-independent effect of ACTH on the *Uts2* mRNA level in the tested cells, with no changes in the *Uts2r* transcript levels. Neither AngII nor K^+^ changed the mRNA levels of the studied genes under these conditions. This is a puzzling observation that suggests that, in vitro, the level of *Uts2r* mRNA in rat adrenocortical cells is independent of ACTH. The lack of significant changes in the *Ust2r* mRNA level observed in our studies, both in vivo and in vitro, may be due to the high constitutive activity of this receptor [43], which is relatively common in other GPCRs.

In this study, it is puzzling that despite the stimulating effect of ACTH on the mRNA level of the *Uts2* gene, the secretion of Uts2 protein by ZG cells (but not ZF/R cells) into the incubation medium was lowered. Similar effects (decreasing Uts2 secretion) were evoked by AngII and K^+^, which, in contrast, did not alter the level of *Uts2* gene expression in the cells studied. An explanation of these differences requires further research.

The next phase of the study involved in vivo experiments conducted to explain the role of Uts2 in the regulation of function of rat adrenals. In adult male rats, both the acute and prolonged administration of ACTH led to an increase in the *Uts2* mRNA level in the adrenal glands, while prolonged administration of DEX did not affect the expression of the studied gene. There were no changes in the *Uts2r* mRNA level in any of these groups. The results of these studies were consistent with the in vitro experiments, in which ACTH affected the mRNA level of *Uts2*, but not *Uts2r*.

In further studies, we focused on the expressions of the *Uts2* and *Uts2r* mRNA levels in the rat adrenal gland in different types of adrenal growth in vivo [44]. Knowing from earlier publications the proangiogenic effects of *Uts2*, we chose a model of adrenal growth known as enucleation-induced adrenal regeneration. In this model, the regeneration of the gland’s cortex is associated with the significant development of the vascular network [45]. During the regeneration of the gland’s cortex, up to day 15 after the surgery, the levels of *Uts2* mRNA did not change in the gland, whereas on days 1 and 5 after the surgery, the level of *Uts2r* transcript decreased. These results suggested that the Uts2/Uts2r system does not significantly affect adrenal cortex growth induced by gland enucleation.

Another model of adrenal growth in vivo is the compensatory growth of the gland induced by hemiadrenalectomy. This is an in vivo adrenal growth model mainly dependent on neural stimuli [44,46,47]. In this type of growth, there is a significant, transient increase in the level of *Uts2* mRNA in the adrenal gland (24 h after surgery), while the mRNA level of its receptor remains unchanged. It seems reasonable to hypothesize that hemiadrenalectomy, which increases blood flow rate in the remaining adrenal gland, leads to an increase in *Uts2* mRNA level in the short term, most likely mediated by factors acting via the paracrine pathway.

Considering the significant sex differences in the structure and function of the rat adrenal cortex [48], we also studied the expressions of *Uts2* and *Uts2r* transcripts in the adrenal glands of adult male and female rats and attempted to explain the role of sex hormones in the regulation of the expression of these genes. Inn rats of both sexes, gonadectomy led to an increase in the mRNA level of *Uts2*, and this effect was prevented by the administration of estradiol or testosterone, respectively, while the mRNA levels of *Uts2* receptor remained unchanged. Explaining these significant and interesting results is difficult. This effect may depend on the action of these hormones at the level of the hypothalamus, as estrogens stimulate the HPA, while androgens inhibit it. What is interesting about this finding is that the described changes in the mRNA level of *Uts2* were not accompanied by changes in the expression of its receptor, which seems to be further evidence of the different regulations of the expression of the two genes (*Uts2* and *Uts2r*). As our results indicate, this phenomenon occurs both in vitro and in vivo.

This study suggests that the regulation of *Uts2* and *Uts2r* mRNA levels differs notably in the adrenal glands of rat. While the *Uts2* mRNA level appears to be mainly dependent on ACTH action, the *Uts2r* transcript is not regulated by this hormone. The studies also suggest that the classic adrenal secretagogues do not have a significant impact on *Uts2/Uts2r* mRNA levels in various types of in vivo gland growth. We also demonstrated that the administration of both estradiol and testosterone lowers the *Uts2* mRNA levels in rat adrenal glands, but the mechanism of this action awaits further investigation.

We are aware that our research has some limitations, mainly because the study’s findings are based exclusively on mRNA levels, which do not necessarily correlate with protein expression changes. In fact, this could be part of the reason for the observed discrepancies between *Uts2* mRNA and Uts*2* secreted protein levels. Future research should include additional tests such as immunoblotting and/or adrenal tissue immunostaining to determine Uts2 and Uts2r protein levels. Moreover, it is essential to note that some of the in vivo experiments in this study included a limited number of biological replicates per group. Further sample collection should be conducted to enhance the robustness of the findings. Furthermore, further research needs to be conducted to address the question of whether the observed lack of stimulation in *Uts2r* mRNA level under ACTH stimulation correlates with its regulation via the paracrine pathway.

## 4. Materials and Methods

### 4.1. Animals and Reagents

The Wistar rats used in our experiments were bred at the Laboratory Animal Breeding Centre, Department of Toxicology, Poznan University of Medical Sciences in Poznan, Poland. The rats were housed under standardized light conditions, following a 14:10 h light/dark cycle with the lights turning on at 6 a.m. The temperature was maintained at 23 °C, with an air humidity of 50–60% and mechanical ventilation providing 8–10 air changes per hour through HEPA filters. The rats had unrestricted access to tap water and were fed a standard diet. Adrenal glands were promptly removed after decapitation. All experimental procedures were conducted in compliance with the protocols approved by the Local Ethics Committee for Animal Studies in Poznan (protocol Nos. 11/2015, 75/2016, and 71/2021). ACTH (Cortrosyn^®^; West Orange, NY, USA) was sourced from Organon Pharmaceuticals, H-89 dihydrochloride hydrate (Merck, B1427, Meguro, Tokyo, Japan) and angiotensin II (Merck, 05-23-0101) were obtained from Merck KGaA (Darmstadt, Germany). The studies were conducted in accordance with the ARRIVE guidelines. The specific details of animal experiments (including study design and sample size) can be found within the sections that describe each experiment.

### 4.2. In Vitro Experiments

#### 4.2.1. Primary Adrenocortical Cell Culture

The previously described protocol of culturing adrenocortical cells obtained from rats was employed in this study [7,49]. Randomly selected male Wistar rats, aged twenty to twenty-two days, were used (*n* = 40). The rats were decapitated; both adrenal glands from each rat were collected and immediately placed in Dulbecco’s modified Eagle’s medium without phenol red (DMEM/F12; Gibco, 17100017, Life Technologies, Carlsbad, CA, USA) before being dissected into smaller pieces using scissors. Subsequently, the tissue fragments were enzymatically digested in DMEM/F12 with type I collagenase (1 mg/mL) for 30 min at 37 °C to obtain a cell suspension. The cell suspension was then filtered through a nylon filter into a test tube and centrifuged at 200× *g* for 10 min. The resulting cells were resuspended in DMEM supplemented with 10% fetal bovine serum (FBS, Merck, F7524) and a solution of antibiotic–antimycotic (1 mL/100 mL; Merck, A5955). The adrenal cells were seeded into 24-well dishes (NUNC Brand Products, Roskilde, Denmark, 142,485) at a density of 1 × 10^4^ cells per well. The cells were incubated under standard conditions of 37 °C and 5% CO_2_. Media exchanges were performed every 24 h. On day 3 of incubation, the medium was supplemented with specific stimulating factors for analysis: ACTH (10^−7^ M), angiotensin II (10^−7^ M), K^+^ (60 mM), or H-89 (10^−5^ M), with six biological replicates per group. After a 2 h incubation period (acute effect) or a 24 h incubation period (prolonged effect), the cells and medium were collected. The medium was used for hormone quantification, while the cells were used for RNA isolation and subsequent qPCR and microarray analyses.

#### 4.2.2. Freshly Isolated Rat Adrenal Cells

After decapitation of 10 adult male and 10 female rats (twenty to twenty-two days old) that were randomly selected, both adrenal glands from each rat were removed, and any adherent fat was dissected. Under a stereomicroscope, the adrenal glands were carefully dissected to isolate the zona glomerulosa (ZG) from the zona fasciculata/reticularis (ZF/R). The isolated cells were then treated following the same procedure as described in the Primary Adrenocortical Cell Culture section, with some differences in the incubation conditions. Specifically, the cells were incubated in a water bath at 37 °C for a duration of 120 min. The culture medium was collected for quantification of aldosterone, corticosterone, and urotensin II. Furthermore, the cells were utilized for RNA extraction to facilitate subsequent analyses. The number of biological replicates was four per group.

#### 4.2.3. RNA Extraction

Total RNA was isolated from the collected cells, specific adrenal sections (ZG, ZF/R, and M), and whole adrenal gland samples. TRI Reagent (Merck KGaA, Darmstadt, Germany, 93289) was employed for the initial RNA extraction process, according to the manufacturer’s protocol. Subsequently, the total RNA underwent purification using columns (Rnasy Mini Kit, Qiagen, Hilden, Germany, 74004). The quantity and purity of the total mRNA were determined using a NanoDrop spectrophotometer (Thermo Fisher Scientific Inc., Waltham, MA, USA). For microarray experiments, 100 ng of total RNA was utilized from each RNA sample, while the remaining RNA was reserved for qPCR analysis.

#### 4.2.4. Reverse Transcription and qPCR

An iScript™ cDNA Synthesis Kit was utilized for reverse transcription (Bio-Rad, Hercules, CA, USA, 1708890). SsoAdvanced Universal SYBR Green Supermix (Bio-Rad, Hercules, CA, USA, 1725271) was used for qPCR. Both reverse transcription and qPCR were performed according to the manufacturer’s protocols. The primers used for quantification of *Uts2* and *Uts2r* were purchased from Bio-Rad (PrimePCR™ SYBR^®^ Green Assay). The primers used for *B2m* quantification were bought from the Laboratory of DNA Sequencing and Oligonucleotide Synthesis, Institute of Biochemistry and Biophysics, Polish Academy of Sciences, Warsaw, Poland. The sequences of *B2m* (NM_012512.2) primers are as follows (5′-3′): CTTGCAGAGTTAAACACGTCA and CTTGATTACATGTCTCGGTC.

#### 4.2.5. Microarray Expression Analysis

The procedure for Affymetrix analysis was conducted as previously described [50]. In brief, cDNA was generated from 100 ng of total RNA using a GeneChip™ WT PLUS Reagent Kit. Subsequently, the cDNA underwent biotin-labeling and fragmentation using an Affymetrix GeneChip^®^ WT Terminal Labeling and Hybridization system. Biotin-labeled fragments of cDNA (5.5 μg) were then subjected to hybridization to an Affymetrix^®^ Rat Gene 2.1 ST Array Strip at 48 °C for a duration of 20 h. Following hybridization, microarrays were washed, stained, and processed using an Affymetrix GeneAtlas Fluidics Station, following the manufacturer’s protocol. The array strips were then scanned using the Imaging Station of a GeneAtlas System. The obtained gene expression data were checked for quality using Affymetrix software (version 2.0). The CEL files generated from the procedure were utilized for downstream data analysis. All data analysis and visualization were performed using the Bioconductor package and R programming language. CEL files were integrated with a corresponding description file. To correct background noise, normalize the data, and generate summarized outcomes, the robust multiarray averaging (RMA) algorithm was employed. Statistical significance analysis of gene expression differences was determined using the t-statistics derived from the empirical Bayes method. The resulting *p*-values were adjusted for multiple comparisons using the Benjamini and Hochberg’s false discovery rate method. For meta-analyses, CEL files were downloaded from the Gene Expression Omnibus database and analyzed in the same manner as the CEL files from the Affymetrix procedure. The only difference was the use of the “rat2302.db” package for annotation data due to the distinct array format utilized in the primary data [51].

#### 4.2.6. Corticosterone, Aldosterone, and Urotensin II Detection

Corticosterone, aldosterone, and urotensin II levels were assessed using an ELISA kit following the instructions provided by the manufacturer (Aldosterone ELISA, cat. No. DE5298 Demeditec Diagnostics GmbH, Kiel, Germany; Corticosterone cat. No. DEV9922 Demeditec Diagnostics GmbH, Kiel, Germany; Urotensin II, cat. No. MBS049343 MyBioSource, San Diego, CA, USA). Absorbance was read with a Biotek—Synergy 2 plate reader (wavelength 450 nm). Quantitative analysis was conducted utilizing a four-parameter logistic curve (4PL) derived from the “drc” Bioconductor package [52].

### 4.3. In Vivo Experiments

#### 4.3.1. The Effects of Acute and Prolonged ACTH Injections on Uts2 and Uts2r mRNA Levels in Rat Adrenal Glands Were Determined

The Wistar male rats used in the study were 12 weeks old, with a body weight ranging from 120 to 150 g, and there were three rats randomly assigned to each experimental group. The groups were as follows: control, ACTH acute effect, ACTH prolonged effect, and DEX. ACTH (5 μg/rat) was administered i.p. 1 h before decapitation in the acute experiment. ACTH, at the same dose, was administered s.c. at hours 0, 12, and 24 in the prolonged experiment. Dexamethasone (DEX; 5 μg/rat) was also administered at hours 0, 12, and 24. Twelve hours after the final injection, the rats were euthanized via decapitation. In the control group, rats were administered a physiological saline solution (0.2 mL/rat) instead of ACTH through injection. Afterward, the rats were decapitated, and their adrenal glands were swiftly removed and used for RNA extraction.

#### 4.3.2. Regeneration Model

The regeneration model is based on the enucleation of both rat adrenal glands under standardized ketamine and xylazine anesthesia. The enucleation was conducted via the dorsal approach using a previously described method [53]. The twenty-one Wistar female rats used in this experiment were 12 weeks old with a body weight ranging from 120 to 150 g. On days 1, 2, 3, 5, 8, and 15 after surgery, rats were decapitated, and their adrenal glands were removed for further analyses. The control group consisted of adrenals obtained from sham-operated rats, specifically collected on day 1 following the sham surgery. Each group comprised three randomly selected rats.

#### 4.3.3. Unilateral Adrenalectomy

The surgical procedure involved the standard administration of ketamine and xylazine anesthesia for both the removal of the left adrenal gland and the sham operation. The hemiadrenalectomy procedure was described elsewhere [54]. Two sham-operated animals were sacrificed 24 h after surgery (control group). The rats from study group were decapitated 24 and 72 h after surgery (experimental groups). The adrenal glands were removed after decapitation and stored for RNA extraction. Each group comprised two randomly selected rats. In this experiment ,six Wistar female rats were used (12 weeks old; 120–150 g).

#### 4.3.4. Effects of Gonadectomy and Sex Hormones’ Supplementation on Uts2 and Uts2r Gene Expressions

The experimental procedures were conducted using adult Wistar rats of both male and female sexes (*n* = 6 male rats and 6 female rats). The rats were 12 weeks old and had a body weight ranging from 120 to 150 g. Rats were anaesthetized and then underwent a gonadectomy or sham surgery. The above-mentioned procedures were described in our previous study [55]. Briefly, two weeks after surgery, rats were administered s.c. with testosterone (Testoviron-Depot, Schering AG, Berlin, Germany, 5 mg/100 g b.w.) or estradiol (Estradiol-Depot, Jenapharm, Jena, Germany, 0.5 mg/100 g b.w.). We used 0.2 mL of sesame oil for the control rats. Rats were decapitated fourteen days later (4 weeks after surgery). Adrenal glands were then promptly removed and stored at −80 °C in RNAlater (Thermo Fisher Scientific Inc., Waltham, MA, USA, AM7021) until subsequent analyses. The following groups were used: male control (sham operated), male gonadectomized, male gonadectomized with testosterone supplementation, female control (sham operated), female gonadectomized, and female gonadectomized with estradiol supplementation. Each group comprised two randomly selected rats.

### 4.4. Statistics

The statistical analysis for comparing two groups was performed using Student’s *t*-test. When comparisons involved more than two groups, a one-way ANOVA was conducted, followed by the post hoc Dunnett’s test. Furthermore, for the comparisons over time, 2-way ANOVA was employed. The statistical significance levels, coefficients of variation, and fold changes are indicated in the graphs.

## Data Availability

Data files regarding the effects of gonadectomy and sex hormone supplementation on the transcriptome profile of rat adrenal glands were deposited in the Gene Expression Omnibus (GEO) repository at the National Center for Biotechnology Information (http://www.ncbi.nlm.nih.gov/geo/, (accessed on 1 July 2023)) under the GEO accession number GEO: GSE93726. Other datasets generated during and/or analyzed during the current study are available from the corresponding author upon reasonable request.
